# Supplementary material for: The Systems Biology Research Tool: evolvable open-source software
Source: BMC Syst Biol. 2008 Jun 29;2:55. doi: 10.1186/1752-0509-2-55 (PMC2446383; doi:10.1186/1752-0509-2-55)
Supplement: Additional file 1 — SBRT Archive. An archive of the current version of the Systems Biology Research Tool. [file 1752-0509-2-55-S1.zip › sbrt-1.4.0/doc/developers_guide/api/sbrt/kernel/math/algebra/MatrixBuilder.html]

MatrixBuilder


|  |  |  |
| --- | --- | --- |
| **Overview** \| **Package** \| **Class** \| **Use** \| **Tree** \| **Deprecated** \| **Index** \| **Help** | |  |
| **PREV CLASS**   **NEXT CLASS** | **FRAMES**    **NO FRAMES**     **All Classes** |  |
| SUMMARY: NESTED \| FIELD \| CONSTR \| METHOD | DETAIL: FIELD \| CONSTR \| METHOD |  |


---


## sbrt.kernel.math.algebra Interface MatrixBuilder<R,C,E>

**Type Parameters:**: `R` - the row index type.: `C` - the column index type.: `E` - the element type.

**All Superinterfaces:**: Matrix<R,C,E>

**All Known Subinterfaces:**: DoubleMatrixBuilder<R,C>, SmdMatrix<R,C>

**All Known Implementing Classes:**: AbstractMdMatrix, AbstractSmdMatrix, HashMatrix, RowOrTreeMatrix, TreeMatrix

---

``` public interface MatrixBuilder<R,C,E> extends Matrix<R,C,E> ```

This interface is used to represent mutable matrices.

**Author:**
:   This interface was written and documented by
    Jeremiah Wright while in the Wagner lab.

---

| **Method Summary** | |
| --- | --- |
| `void` | `removeColumn(java.lang.Object columnIndex)`             Removes the column corresponding to the provided column index. |
| `void` | `removeRow(java.lang.Object rowIndex)`             Removes the row corresponding to the provided row index. |
| `<X extends R,Y extends C,Z extends E> void` | `setElement(X rowIndex, Y columnIndex, Z elementValue)`             Sets the element at the provided row and column indices to the one provided. |
| `void` | `setElements(Matrix<? extends R,? extends C,? extends E> m)`             Sets the corresponding elements of this matrix to those of the provided matrix. |

**Methods inherited from interface sbrt.kernel.math.algebra.Matrix**

`columns, equals, getColumn, getColumnIndices, getElement, getRow, getRowIndices, hashCode, isColumnIndex, isEmpty, isRowIndex, rows, toString`

**Method Detail**

### removeRow

```
void removeRow(java.lang.Object rowIndex)
```

:   Removes the row corresponding to the provided
    row index.

    :   **Parameters:**: `rowIndex` - the index of the row to be removed.

---


### removeColumn

```
void removeColumn(java.lang.Object columnIndex)
```

:   Removes the column corresponding to the provided
    column index.

    :   **Parameters:**: `columnIndex` - the index of the column to be removed.

---


### setElements

```
void setElements(Matrix<? extends R,? extends C,? extends E> m)
```

:   Sets the corresponding elements of this matrix to
    those of the provided matrix.

    :   **Parameters:**: `m` - the matrix whose elements are to be copied into this matrix.

---


### setElement

```
<X extends R,Y extends C,Z extends E> void setElement(X rowIndex,
                                                      Y columnIndex,
                                                      Z elementValue)
```

:   Sets the element at the provided row and column indices
    to the one provided.

    :   **Type Parameters:**: `X` - a type extending the primary row index type.: `Y` - a type extending the primary column index type.: `Z` - a type extending the primary element type. **Parameters:**: `rowIndex` - the row index.: `columnIndex` - the column index.: `elementValue` - the element.


---


|  |  |  |
| --- | --- | --- |
| **Overview** \| **Package** \| **Class** \| **Use** \| **Tree** \| **Deprecated** \| **Index** \| **Help** | |  |
| **PREV CLASS**   **NEXT CLASS** | **FRAMES**    **NO FRAMES**     **All Classes** |  |
| SUMMARY: NESTED \| FIELD \| CONSTR \| METHOD | DETAIL: FIELD \| CONSTR \| METHOD |  |


---
